# Stress Echocardiography Revisited: Toward Improving Patient Selection and Clinical Decision‐Making

**DOI:** 10.1111/echo.70186

**Published:** 2025-08-18

**Authors:** Silviu Stanciu, Mariana Floria, Diana‐Elena Iov, Daniela Maria Tanase

**Affiliations:** ^1^ Bucharest Faculty of Pharmacy, Internal Medicine University of Medicine and Pharmacy Carol Davila Bucharest Romania; ^2^ Central Military University Emergency Hospital Bucharest Romania; ^3^ I Medical Clinic St Spiridon Iasi Emergency Clinical Hospital Iași Romania; ^4^ Internal Medicine Grigore T Popa University of Medicine and Pharmacy Iasi Iași Romania; ^5^ St Spiridon Iasi Emergency Clinical Hospital Iași Romania; ^6^ UMF Iasi Iași Romania

**Keywords:** echocardiography, ischemia, myocardial viability, stress

1

Nowadays stress echocardiography is globally adopted in clinical practice. The European Society of Cardiology and the American College of Cardiology/American Heart Association recommend SE as the first‐line option for investigation of suspected ischemic heart disease (IHD) [1, 2]. Since the first use of SE in identifying myocardial ischemia and/or myocardial viability, this ultrasound technique has evolved greatly. For example, triple imaging SE means the assessment of the regional wall motion (kinetics), coronary flow reserve, and left ventricular elastance. In addition, two‐dimensional and three‐dimensional speckle tracking echocardiography are utilized to measure regional changes in longitudinal strain and area strain through their polar projection [3]. These ultrasound approaches allow a more detailed evaluation of the locations and severity of myocardial damage. Analyzing strain can uncover subclinical cardiac dysfunction or remodeling of cardiomyocytes. As the myocardium undergoes structural and functional remodeling, cardiac strain diminishes, making it a valuable marker for assessing disease progression. Nowadays, beside IHD, currently indications of SE are^3^: (i) in heart failure for prediction of contractile reserve and the response to medical therapy in cardiac resynchronization therapy, for B lines stress detection, for diastolic heart failure assessment; (ii) in dilated and hypertrophic cardiomyopathy for genetic stress echo; (iii) in valvular heart diseases for transvalvular or surgical aortic valve replacement; (iv) in congenital heart diseases for right ventricular contractile reserve; (v) for pulmonary hypertension, and (vi) in high altitude and extreme physiology (SE outdoor). Therefore, SE is evolving very much, having now a diagnostic, therapeutic decision‐making, and prognostic role in many heart diseases.

Dobutamine is a synthetic catecholamine that enhances myocardial contractility and is often used in assessing cardiac function in patients with IHD. Various doses of dobutamine can have differing effects on myocardial viability; lower doses may improve contractility without significantly increasing oxygen demand, while higher doses may lead to arrhythmias and increased myocardial oxygen consumption. In patients with IHD, the use of β‐blockers can counteract some of the adverse effects of increased heart rate and myocardial oxygen demand induced by dobutamine. While dobutamine SE can help identify viable myocardium, the presence of β‐blockers may modify the functional response, enhancing the protective effects on cardiac workload and potentially improving outcomes in patients with heart failure due to IHD. The recently published study conducted by Li et al. [4] investigated the prognostic value of varying doses of dobutamine SE in evaluating myocardial viability in patients with IHD. Three dosing protocols of dobutamine—low, medium, and high—were assessed. The researchers examined the impact of administering 5 mg of intravenous metoprolol within 1 minute after the dobutamine SE procedure on both adverse drug reactions and myocardial viability in these patients. The medium‐dose group demonstrated the highest sensitivity, specificity, and overall accuracy in identifying myocardial viability, outperforming both the low‐ and high‐dose groups. These findings provide valuable guidance for optimizing dobutamine dosing in clinical settings, allowing for real‐time adjustments that can enhance the precision and reliability of viability assessments. Moreover, the study found that β‐blockers, such as metoprolol, can significantly improve diagnostic outcomes in IHD patients. By stabilizing cardiac function, β‐blockers contribute to more accurate assessments of myocardial performance, thereby increasing both the sensitivity and specificity of viability detection [4]. It is well known that, when formulating clinical strategies, physicians should carefully consider individual patient symptoms and treatment needs to ensure a balance between therapeutic efficacy and safety. While dobutamine is known to be effective in improving myocardial viability, this study observed, as expected, a higher incidence of arrhythmias and other cardiovascular events in the high‐dose group. This underscores the necessity of vigilant monitoring for adverse effects when administering higher doses and suggests that such side effects may limit the broader clinical application of high‐dose protocols. Overall, this study emphasizes the importance of selecting the appropriate dobutamine dose and highlights the beneficial role of β‐blockers in enhancing diagnostic accuracy for myocardial viability in patients with IHD [4]. It is a new small step of SE toward improving patient selection and clinical decision‐making.

The viable myocardium, often characterized by preserved wall motion or recovery potential, correlates with better outcomes postrevascularization, such as improved systolic function and reduced mortality. Incorporating myocardial viability into clinical practice not only enhances risk stratification in patients with heart failure but also guides therapeutic decision‐making, ultimately improving patient prognosis [5]. Dobutamine SE evaluates contractile reserve, and color Doppler tissue imaging can assess changes in myocardial motion and deformation. Given its accessibility, lack of ionizing radiation, and ability to provide valuable functional insights, myocardial viability continues to be an essential aspect of cardiac imaging and management strategies [6]. By combining the peak global longitudinal strain, global work index, and peak global myocardial work efficiency, the accuracy of an abnormal dobutamine ES can be improved [7].

According to the ABCDE protocol of SE, the assessment of the regional wall motion and regional perfusion using ultrasound‐contrast agents is the main step A [5]. The next step B means diastolic function evaluation and the pulmonary B‐lines identification. The analysis of left ventricular contractile and preload reserve with volumetric echocardiography represents step C. Doppler‐based coronary flow velocity reserve in the left anterior descending coronary artery is step D, followed by ECG‐based heart rate reserve in nonimaging step E. This ABCDE protocol allows comprehensive risk stratification of patients with IHD. The type of ischemic cascade triggered by SE is specific for the location and implicitly the cause of the angina in chronic coronary syndrome (epicardial, microvascular, vasospasm) [5]. These different endotypes are unmasked by SE patterns.

Myocardial viability by dobutamine SE or late gadolinium enhancement MRI is a crucial tool in assessing cardiac function and determining the appropriateness of revascularization strategies in patients with IHD [3]. These noninvasive imaging technique focuses on identifying areas of the myocardium that remain viable despite ischemic conditions or previous infarctions [3, 6, 7, 8]. In myocardial viability assessment, techniques such as magnetic resonance imaging (MRI) or single positron emission computer tomography (SPECT) demonstrate the highest sensitivity, specificity, and accuracy [3]. Magnetic resonance imaging late gadolinium enhancement is the gold standard for functional assessment of cardiac function, myocardial viability, quantitative flow evaluation, and tissue characterization, offering excellent soft‐tissue contrast. It provides excellent sensitivity for detecting viable myocardium while minimizing false positives associated with nonviable regions. Thanks to the high temporal and spatial resolution, MRI perfusion imaging can accurately assess myocardial ischemia, quantify myocardial blood flow, and detect microvascular dysfunction [9]. Positron emission tomography using metabolic tracers like fluorodeoxyglucose enhances specificity by distinguishing between viable and nonviable myocardium based on metabolic activity [3, 9]. Both modalities have shown accuracy rates exceeding 90%, making them preferable for determining treatment strategies in patients with IHD [5]. Coronary computed tomography angiography allows the corroboration of the anatomical data with hemodynamic evaluation through noninvasive fractional flow reserve computation or stress myocardial perfusion analysis [9].

In real‐world settings, the level of myocardial ischemia assessed by clinicians during SE accurately predicts the risk of future cardiovascular events within 5 years [10]. Classifying a stress echocardiogram as negative for individuals without a history of coronary artery disease effectively identifies patients who face no greater risk of cardiovascular events than the general background risk over the same timeframe [10, 11]. It was shown that the “warranty period” of SE lasts up to 4 years for individuals with a prior diagnosis of coronary artery disease, while for those without any documented coronary conditions, this period extends to at least 5 years. Compared with other functional imaging tests, coronary computed tomography angiography has a warranty period of 2 years, according to the latest guidelines from the American College of Cardiology [10, 11].

Although SE is the most widely available functional imaging test in the diagnosis and management of IHD, MRI, SPECT, coronary computed tomography angiography or myocardial perfusion scintigraphy are other imaging modalities which are very useful in the assessment of IHD in order to improve patient selection and clinical decision‐making. These imaging modalities allow examining coronary artery and especially myocardial status as a contiguous spectrum from viable to partially viable myocardium with varying degrees of subendocardial scar and nonviable myocardium with predominantly transmural scar, the therapeutic and prognostic determinants in IHD. A combination of these approaches may provide the most comprehensive assessment; the choice of imaging modality often depends on the clinical scenario, availability of technology, and specific patient characteristics. However, SE continues to be a fundamental tool for appropriate assessment of IHD in clinical settings by combining few imaging techniques. The main challenge for SE remains how diagnostic pathways can be translated into management decisions. Therefore, the study published by Li et al. [4] contributes to the refinement of patient selection and clinical decision‐making in chronic coronary syndrome.
